# Associations Between Nutritional Intake, Body Composition, Menstrual Health, and Performance in Elite Female Trail Runners

**DOI:** 10.3390/jfmk10040482

**Published:** 2025-12-17

**Authors:** Nil Piñol-Granadino, Marta Carrasco-Marginet, Silvia Puigarnau, Javier Espasa-Labrador, Álex Cebrián-Ponce, Fabrizio Gravina-Cognetti, Maria Darder-Terradas, Joan Solé-Fortó

**Affiliations:** 1 National Institute of Physical Education of Catalonia (INEFC), Av. De l’Estadi 12-22, 08038 Barcelona, Spain; nilpigra@gmail.com (N.P.-G.); spuigarnau@gencat.cat (S.P.); fgravina@gencat.cat (F.G.-C.); jsolef@gencat.cat (J.S.-F.); 2 INEFC-Barcelona Research Group on Sport Sciences (GRCEIB), Av. De l’Estadi 12-22, 08038 Barcelona, Spain; javierespasa12@gmail.com (J.E.-L.); acebrianponce@gmail.com (Á.C.-P.); 3 Catalan School of Kinanthropometry (ECC-INEFC), Av. De l’Estadi 12-22, 08038 Barcelona, Spain; 4 Faculty of Computer Science, Multimedia and Telecommunications, Open University of Catalonia (UOC), Rambla del Poblenou 154-156, 08018 Barcelona, Spain; mariadardert@gmail.com

**Keywords:** female athletes, relative energy deficiency in sport, low energy availability, menstrual dysfunction, sports medicine, ultra-endurance running, anthropometry, endurance sports

## Abstract

**Background**: This study examined nutritional intake, body composition, menstrual health, and performance in elite female trail runners. **Methods**: A cross-sectional multivariate analysis was conducted on 35 athletes (14 eumenorrheic, 21 amenorrheic/oligomenorrheic). Nutritional intake was assessed through 7-day and 24 h food records; anthropometry followed ISAK standards; performance was evaluated via ITRA and UTMB rankings. Statistical analyses included *t*-tests, MANCOVA, regression models, and Random Forest, adjusting for body composition and covariates. **Results**: Although energy availability (EA) did not differ significantly between groups, 94.3% of athletes had clinically low EA (<30 kcal/kg FFM/day). Amenorrheic athletes consumed more simple carbohydrates (21.8 ± 5.7% vs. 17.2 ± 3.1%), protein (2.5 ± 0.6 vs. 1.7 ± 0.2 g/kg/day), fiber, and lipids, while eumenorrheic athletes consumed more complex carbohydrates (129.7 ± 27.0 vs. 82.5 ± 33.3 g/day) and most vitamins. Both groups had inadequate calcium and iron intake. Low EA was moderately associated with an ectomorphic somatotype (r = 0.418). Performance negatively correlated with simple carbohydrates (r = −0.624) and positively with complex carbohydrates, total energy, protein, polyunsaturated fats, and zinc (r = 0.300–0.580). No significant performance differences were found between menstrual status groups. **Conclusions**: Menstrual irregularities did not affect performance, but nutritional patterns strongly influenced both performance and energy availability. Personalized nutrition strategies are essential for optimizing performance and safeguarding health in elite female trail runners.

## 1. Introduction

Until relatively few years ago, research in female physiology has primarily focused on reproduction, ignoring the non-reproductive functions of ovarian hormones such as estrogen and progesterone, whose cyclical fluctuations are regulated by pituitary gonadotropins—follicle-stimulating hormone (FSH) and luteinizing hormone (LH)—and not only ensure menstrual and reproductive function but also modulate bone, cardiovascular, thermoregulatory, neuromuscular, and metabolic processes that may influence endurance performance in female athletes [1,2,3,4]. For many years, women have rarely been included as participants in sports science research, and even when they are, the applied methodologies often fail to consider their specific physiological needs [5]. A recent analysis of sports medicine and exercise science publications found a marked underrepresentation of female participants, with only about 4% to 13% of articles focused exclusively on women [6,7]. Even when women are included in sports research, their hormonal fluctuations are often overlooked, or tests are frequently conducted during menstrual phases of low hormone levels to minimize potential hormonal interference [7,8]. These research gaps and omissions can be attributed to several factors [5]: a lack of progress in the development of women’s sports compared to men’s (often accompanied by discouraging attitudes toward female competitive participation), a shortage of researchers with expertise in female endocrinology and exercise physiology, the greater financial and time investment required for high-quality research on female athletes, and significantly less funding for women-focused research projects compared to those focused on men. These historical limitations have resulted in a dearth high-quality evidence to properly guide female athletes in their sporting pursuits.

In competitive sports, female athletes are particularly vulnerable to adverse health effects often linked to low energy availability—LEA [9]. Energy availability is defined as the amount of energy remaining for physiological processes after subtracting the energy expended during exercise [10]. When energy availability is insufficient, critical processes such as cellular maintenance, thermoregulation, and reproductive function can be compromised [11]. In many cases, this state occurs even in otherwise healthy athletes due to a lack of awareness of their daily energy requirements relative to training loads and competitive demands. However, if this energy deficiency persists over time, it can lead to a number of adverse consequences, including menstrual and reproductive dysfunctions [12], compromised bone health [11], impaired vitamin D absorption [13], immune dysfunctions [14], reduced protein synthesis [15], increased cardiovascular risk [16], and diminished athletic performance [17]. In the most extreme cases, particularly among athletes who deliberately and dramatically restrict their caloric intake, dangerous eating behaviors can emerge, potentially progressing to eating disorders [18].

In recent decades, the exponential rise in endurance and ultra-endurance events has led to a surge in the popularity of trail running—TR—[19]. However, research on this discipline remains limited compared to other sports, and studies focusing specifically on female trail runners are even scarcer [20,21,22,23]. Although some studies indicate that sex-based hormonal differences can influence the performance of female trail runners [19,24,25], other biological factors differentiating female and male athletes remain insufficiently explored in this context [20,21,26,27]. In terms of sports nutrition, only a few studies to date have analyzed female trail runners’ nutritional intake and hydration strategies during competition [28,29,30,31], and no study has yet thoroughly examined their daily dietary habits.

This lack of research is concerning, since elite female trail runners—due to their high training loads and competitive demands—constitute a high-risk population for developing metabolic and hormonal disorders. In fact, a significant prevalence of secondary amenorrhea and oligomenorrhea has been reported in this population, underscoring the need to characterize their dietary habits and body composition with respect to menstrual cycle status [32]. This study aims to provide a detailed characterization of the nutritional patterns, body composition profile, and athletic performance of international-level female trail runners, analysing how nutrition and body composition relate to both athletic performance and menstrual cycle status, as well as examining the influence of the menstrual cycle on athletic performance.

## 2. Materials and Methods

### 2.1. Ethical Approval

This study was approved by the Ethics Committee for Clinical Research of the Catalan Sports Council (0099/S690/2013). All participants provided written informed consent and received detailed information about the procedures. To ensure the protection of participants’ rights, the study complied with the ethical principles of the Declaration of Helsinki, the Nuremberg Code, and the Belmont Report, as well as all applicable national and regional regulations.

### 2.2. Trial Design

This investigation was designed as a descriptive, comparative, and correlational cross-sectional study using a non-probabilistic convenience sample. Participants were stratified into two groups according to menstrual status (eumenorrheic vs. secondary amenorrheic/oligomenorrheic), allowing for both group-based comparisons and multivariate exploration. Four main assessment domains were included, each composed of multiple independent variables: (1) Nutritional assessment—encompassing metrics related to total energy intake, energy availability, macronutrient distribution, fiber, and intake of key micronutrients; (2) Anthropometric assessment—including anthropometric characteristics, body composition estimates, and somatotype components; (3) Menstrual health assessment—evaluating indicators of reproductive function based on questionnaire responses; and (4) Performance assessment—based on international competitive rankings. The design allowed for the identification of differences between menstrual status groups across all domains, while also supporting the examination of interrelationships among nutritional, physiological, and performance-related variables. Menstrual status was modeled as the main outcome of interest to determine which factors were most strongly associated with menstrual dysfunction in this athletic population. Additionally, the influence of nutritional intake on sports performance was analyzed, accounting for relevant anthropometric characteristics. The analytical framework incorporated both group-level comparisons and integrated multivariate modeling, with consideration of covariates and interactions across variables, enabling a comprehensive understanding of how energy availability, dietary habits, and physical characteristics relate to menstrual health and athletic performance in elite female trail runners.

### 2.3. Participants

A total of 35 elite female trail runners, all members of the Spanish national team, voluntarily participated in the present study. To ensure adequate statistical power, an a priori sample size estimation was performed using G*Power v3.1.9.6, based on a two-group comparison with an assumed effect size of d = 0.85, a power of 72.0%, and a significance level of α = 0.05. The analysis indicated a minimum required sample size of *n* = 32 (*n* = 11 for group 1 and *n* = 21 for group 2). Recruitment was conducted in collaboration with the Spanish Federation for Mountain and Climbing Sports (FEDME). At the time of data collection, all female athletes were in the general preparatory phase of their annual training cycle and had competed at the international level either during the current or previous competitive season. General characteristics and training backgrounds of the participants are presented in Table 1, according to the final grouping based on menstrual status: eumenorrheic (*n* = 14) and amenorrheic/oligomenorrheic (*n* = 21).

Inclusion criteria required participants to be adult female athletes actively engaged in structured training and international competition. Exclusion criteria comprised a prior diagnosis of anorexia nervosa (as defined by DSM-V), diabetes mellitus, corticosteroid therapy, hyperparathyroidism, gastrointestinal conditions involving malabsorption, or other disorders potentially affecting energy metabolism. To minimize confounding effects on body composition, athletes were also excluded if they had performed physical activity within 48 h prior to testing or reported the use of dietary supplements known to alter tissue dynamics.

### 2.4. Procedures

#### 2.4.1. Nutritional Assessment

Each participant completed a dual dietary assessment: a 7-day food diary and a 24 h recall, capturing all food and beverage intake. Multi-day records are widely used in sports nutrition to account for intake variability [33], with 3–7 days considered a practical and valid window for athletes [34]. To improve accuracy, participants received visual guides with portion-size images of common foods. Such aids enhance the validity of self-reported intake [35]. Individual instructions emphasized precise reporting of quantities, preparation methods, and timing. Supplement and medication use (e.g., iron or oral contraceptives) was also recorded, given their dietary relevance [36]. All records were reviewed one-on-one by a sports nutritionist to resolve ambiguities and ensure completeness—an approach that minimizes reporting errors [37]. Food diaries were analyzed using CESNID Nutritional Analysis Software^®^ (version 1.0) [38], developed by the University of Barcelona (Barcelona, Spain), based on a Spanish-specific food composition database. Nutrient and energy intakes were compared to recommended guidelines for athletes [39]. To assess energy adequacy, total intake was compared to individualized requirements: basal metabolic rate was calculated via the Harris-Benedict equation with a 1.3 activity factor for light daily activity [40], and training-related expenditure was added using MET values from the Compendium of Physical Activities [41]. Total energy intake was expressed relative to each athlete’s estimated needs, and all nutritional variables were reported as percentages of optimal recommendations [39], facilitating the evaluation of dietary adequacy and potential performance or health implications.

#### 2.4.2. Anthropometric Assessment

A full anthropometric profile was assessed in accordance with the International Society for the Advancement of Kinanthropometry (ISAK) standards [42]. All measurements were conducted by a single accredited anthropometrist (ISAK Level 3) who was part of the research team, ensuring consistency and minimizing inter-evaluator variability. The protocol included basic measurements (body mass, stretch stature, sitting height, and arm span); eight skinfolds (triceps, subscapular, biceps, iliac crest, supraspinale, abdominal, thigh, and calf); girths (head, neck, arm relaxed, arm flexed and tensed, forearm, wrist, chest, waist, hip, thigh 1 cm gluteal, thigh middle, calf, and ankle); lengths and heights (acromiale–radiale, radiale–stylion, midstylion–dactylion, iliospinale height, trochanterion height, trochanterion–tibiale laterale, tibiale laterale height, foot, and tibiale mediale–sphyrion tibiale); and breadths (biacromial, antero-posterior abdominal depth, biiliocristal, transverse chest, antero-posterior chest depth, humerus, bi-styloid, femur, and bimalleolar). All anthropometric variables were measured twice using non-consecutive measurements. A third measurement was taken if the difference between the first two exceeded 1% for all variables, or 5% in the case of skinfolds [42]. Final values were calculated as the mean of the first two measurements, or the median of the three if a third was required. The intra-evaluator technical error of measurement was notably low: 0.021% for basic measurements, 1.37% for skinfolds, 0.83% for girths, 0.32% for lengths and heights, and 0.45% for breadths and depths, confirming a high level of precision. Anthropometric instruments included a Seca 220^®^ telescopic stadiometer (Seca GmbH & Co. KG, Hamburg, Germany) (range: 85–200 cm; precision: 0.1 cm); a pre-calibrated Seca 710^®^ mechanical scale (Seca GmbH & Co. KG, Hamburg, Germany) (capacity: 200 kg; precision: 0.05 kg); a Realmet© anthropometric tape (Realmet, Nottingham, UK) (precision: 0.1 cm); a Realmet© segmometer (Realmet, Nottingham, UK) (precision: 0.1 cm); a Harpenden^®^ skinfold caliper (Baty International, Burgess Hill, UK) (range: 0–90 mm; precision: 0.2 mm; constant pressure: 10 g/mm^2^); and Holtain^®^ large and small sliding bone breadth calipers (Holtain Ltd., Crymych, UK) (range: 0–25 cm; precision: 0.1 cm). From the full ISAK-compliant profile, several derived indicators were calculated. Body proportionality indices and muscular cross-sectional areas were used to assess structural relationships among anthropometric dimensions. Body composition estimates—including fat mass [43], muscle mass [44], and bone mass [45]—were computed using validated anthropometric equations for athletic populations. Additionally, the Heath-Carter somatotype method [46] was used to determine individual morphological profiles, expressed in terms of endomorphy (relative fatness), mesomorphy (muscularity), and ectomorphy (linearity).

#### 2.4.3. Menstrual Health Assessment

Menstrual health was assessed through a two-step approach. First, each athlete completed a detailed ad hoc menstrual history questionnaire, developed based on established literature definitions to classify their cycle status as eumenorrheic or amenorrheic/oligomenorrheic. Amenorrhea was conservatively defined as the absence of menstruation for over 90 days, in accordance with established clinical criteria [47]. Oligomenorrhea was defined as menstrual cycles longer than 35 days (approximately 5–7 periods per year), often associated with hypothalamic-pituitary-ovarian axis dysfunction in female athletes [48]. Second, an adapted version of the Low Energy Availability in Females Questionnaire (LEAF-Q) was administered, focusing exclusively on the reproductive and injury-related items. This adaptation was chosen to target key indicators of Relative Energy Deficiency in Sport (RED-S) [49], while avoiding redundancy, as gastrointestinal symptoms were assessed more precisely through comprehensive dietary intake data obtained from the 7-day food diary and 24 h recall. The LEAF-Q’s proven sensitivity, ease of administration, and validation across athletic populations supported its use in this elite endurance cohort [10,50]. This approach allowed a focused and efficient assessment of menstrual disturbances and injury history relevant to RED-S risk without overlap with detailed nutritional data.

#### 2.4.4. Performance Assessment

Athletic performance was assessed using scores from two internationally recognized ranking systems: the ITRA^®^ Performance Index (International Trail Running Association) and the UTMB^®^ Index (Ultra-Trail du Mont-Blanc). Both systems are widely used in the trail running field to quantify competitive level, assigning standardized scores ranging from 0 to 1000 points through proprietary algorithms developed by their respective organizations. These indices integrate objective parameters such as race distance, elevation gain, and finishing time, alongside contextual factors like terrain type and environmental conditions. The algorithms draw on extensive databases of historical race results, enabling normalization and cross-event comparability. To increase the robustness and representativeness of the performance assessment, both scores were combined by calculating their arithmetic mean. This approach yielded a coherent and reliable indicator of each athlete’s competitive status.

### 2.5. Statistical Analysis

The distribution of each variable was examined using the Kolmogorov–Smirnov test to assess normality. Descriptive statistics were reported as means and standard deviations. Between-group comparisons (eumenorrheic vs. amenorrheic/oligomenorrheic) were conducted using either the independent samples Student’s *t*-test or the Mann–Whitney U test, depending on whether the assumptions of normality were met. Effect sizes were calculated using Cohen’s d, with the following thresholds: 0.21–0.49 (small), 0.50–0.70 (moderate), and ≥0.71 (large). For correlation coefficients, effect size thresholds were interpreted as |r| = 0.10–0.29 (small), |r| = 0.30–0.49 (moderate), and |r| ≥ 0.50 (large) [51]. Associations between anthropometric parameters and indicators of LEA were analyzed using Pearson or Spearman correlation coefficients, depending on data distribution. Additionally, Random Forest models were implemented as a non-linear classification technique, incorporating bootstrap validation (100 iterations) and a train/test data partitioning strategy to enhance model robustness. To account for potential confounding factors and to evaluate adjusted group differences, a Multivariate Analysis of Covariance (MANCOVA) was applied. The relationship between nutritional intake and athletic performance was explored using correlation matrices, stepwise multiple linear regression models, and univariate regression analyses. To reduce the likelihood of Type I error, *p*-values were corrected using the False Discovery Rate (FDR) procedure. Multicollinearity was assessed using the Variance Inflation Factor (VIF), and partial correlations were computed with adjustment for covariates. All statistical procedures were performed in RStudio (version 4.4.3, RStudio, PBC, Boston, MA, USA), with the alpha level for statistical significance set at *p* ≤ 0.050.

## 3. Results

### 3.1. Nutritional Results

This section presents the results related to the nutritional status of the female trail runners included in the study (Table 2).

No statistically significant differences were found in energy availability between eumenorrheic and amenorrheic/oligomenorrheic athletes. However, 94.3% of participants presented values below 30.0 kcal/kg, the threshold widely recognized for clinical LEA [52]. Regarding carbohydrate intake, total carbohydrate consumption—both absolute and relative to body mass—did not differ significantly between groups. Nevertheless, eumenorrheic athletes reported significantly higher intake of complex carbohydrates, whereas athletes with menstrual dysfunction consumed significantly more simple carbohydrates. Protein intake was higher in the amenorrheic/oligomenorrheic group across all measures: total grams per day, grams per kilogram of body mass, and percentage of energy intake. Similarly, fiber intake was significantly greater in this group. The total lipid intake (g/kg/day) was significantly higher in amenorrheic/oligomenorrheic athletes compared to eumenorrheic athletes.

However, eumenorrheic athletes had a higher intake of saturated and monounsaturated fats. Polyunsaturated fats and cholesterol intake did not differ significantly. Vitamin intake differed by menstrual status. Eumenorrheic athletes consumed significantly higher amounts of vitamins B2, B6, C, D, and E, while no group differences were observed for vitamins B1 and B12. Mineral intake also varied: calcium intake was significantly higher in eumenorrheic athletes, while differences in iron and zinc intake were not statistically significant. MANCOVA, adjusted for body composition, revealed a significant overall effect of menstrual status on the nutritional profile (Wilks’ Lambda = 0.127, *p* = 0.005). This effect remained particularly strong for complex carbohydrate (η^2^ = 0.64; *p* < 0.001) and vitamin B12 intake (η^2^ = 0.13; *p* = 0.046). Regarding the relationship between nutrition and sports performance (ITRA-UTMB score), most nutritional variables showed negative correlations. The strongest and most practically relevant association was observed for simple carbohydrate intake (r = −0.624, *p* <0.001), which represents a strong, statistically significant correlation. Other variables showed statistically significant moderate negative correlations (r = −0.300 to −0.500), including total energy intake (*p* = 0.010), carbohydrate intake per kg of body weight (*p* = 0.0123), and several micronutrients (vitamins B2 (*p* = 0.017), B6 (*p* = 0.001), C (*p* = 0.024), D (*p* = 0.004), calcium (*p* = 0.003), iron (*p* = 0.004)). Although these moderate correlations may have some practical relevance, other associations (e.g., energy availability, protein intake per kg, cholesterol, zinc) either did not reach statistical significance (*p* ≥ 0.050) or were of weak magnitude (|r| < 0.30) and were therefore interpreted as having limited practical impact despite their direction of effect. Remaining variables showed weak correlations (r between −0.300 and 0.300) and no statistical significance. A multiple linear regression model, refined by stepwise selection, explained approximately 96% of the variability in performance. Positive associations were found for energy, complex carbohydrates, protein, polyunsaturated fats, and zinc, while negative associations were found for simple carbohydrates, saturated fats, cholesterol, fiber, and energy availability. These results were further supported by individual regression models. Partial correlation analyses, adjusted for body composition, indicated that lower energy availability remained associated with higher performance levels, independent of anthropometric covariates.

### 3.2. Anthropometric Results

The anthropometric characteristics of the athletes, categorized by menstrual cycle status, are detailed in Table 3.

No statistically significant differences were observed in general body dimensions, including body mass, stature, sitting height, and arm span, indicating comparable structural size across groups. In contrast, significant between-group differences were observed in skinfold measurements. Athletes with secondary amenorrhea or oligomenorrhea presented significantly lower values in the subscapular and abdominal skinfolds (*p* < 0.050), with a non-significant trend toward lower values across the remaining sites. The sum of eight skinfolds was also lower in this group, although the difference did not reach statistical significance. Regarding girths, significantly smaller hip, mid-thigh (1 cm below the gluteal fold), and calf circumferences were observed in the amenorrheic/oligomenorrheic group (*p* < 0.050). No significant differences were found in upper limb or trunk perimeters. Segmental lengths and heights, as well as transverse and sagittal diameters, did not differ significantly between groups, except for the transverse thoracic diameter, which was greater in the eumenorrheic athletes (*p* = 0.012). No statistically significant differences were found in proportionality indices such as BMI or WHR, nor in muscle area estimations, although eumenorrheic athletes showed higher non-significant values in arm, thigh, and total muscle cross-sectional areas.

With regard to body composition, the amenorrheic/oligomenorrheic group showed lower fat mass percentages and higher skeletal muscle mass values, in line with the differences observed in skinfolds and girths. Estimated bone mass, calculated using the Rocha and Withers equations, did not show significant differences between groups. Finally, somatotype analysis revealed no statistically significant differences, although a trend was observed toward higher ectomorphic values and lower endomorphic and mesomorphic components in athletes with menstrual dysfunction. Among all anthropometric variables, only the ectomorphic component showed a statistically significant moderate correlation with LEA *r* = 0.418 *p* = 0.012, which may be of practical relevance in indicating that leaner body builds are more susceptible to low energy availability. Multivariate analysis, adjusted for body composition, revealed a statistically significant overall effect of menstrual cycle status on the anthropometric profile (Wilks’ Lambda = 0.403; *p* < 0.001). The most discriminant variables included the subscapular and abdominal skinfolds, hip and thigh circumferences, and muscle area parameters. Random Forest modeling yielded a sensitivity of 100% and a specificity of 50%, with a moderate agreement (kappa = 0.571), indicating variable classification performance based on morphological traits. No statistically significant associations were found between any anthropometric variable and the ITRA-UTMB performance score. Correlation coefficients remained below the moderate threshold (|r| < 0.350), and *p*-values were above 0.050. Individual linear regression models confirmed the absence of statistically significant predictors among the anthropometric variables analyzed.

### 3.3. Sports Performance Ranking According to Menstrual Cycle

**Figure 1:**
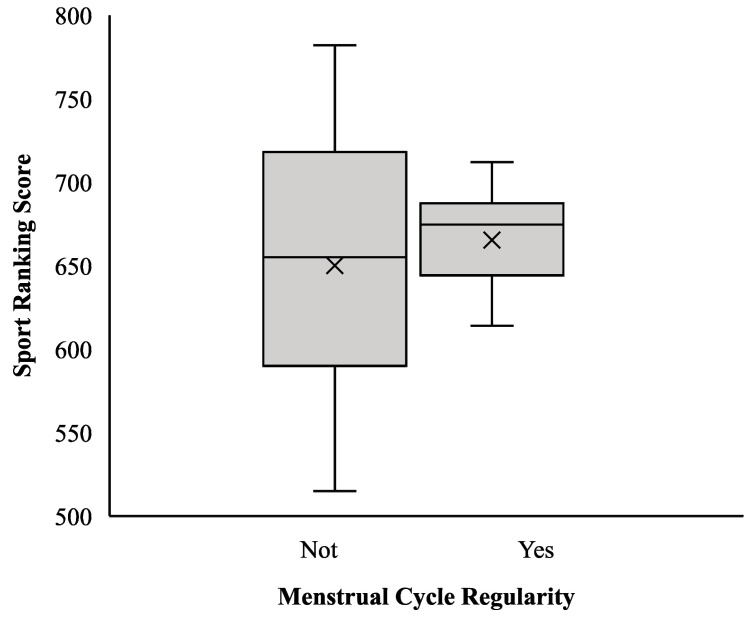
Sport Ranking Score According to Menstrual Cycle Regularity.

No statistically significant differences were observed in sports performance, as measured by the ITRA-UTMB composite ranking, between athletes with regular menstrual cycles (eumenorrheic) and those reporting menstrual irregularities (oligomenorrhea or secondary amenorrhea). The mean performance score was 664 points (median = 676; interquartile range [IQR] = 653–695) in the eumenorrheic group and 653 points (median = 655; IQR = 592–726) in the irregular cycle group. The distribution of scores in the eumenorrheic group showed less variability, with a narrower IQR and smaller overall range compared to the irregular cycle group. Figure 1 presents the corresponding box plot illustrating these differences in central tendency and dispersion. However, none of these comparisons reached statistical significance (*p* > 0.050).

## 4. Discussion

To the best of our knowledge, this is the first study to simultaneously examine the relationships between dietary intake, anthropometric characteristics, menstrual cycle status, and competitive performance in elite female trail runners. By including a standardized performance indicator based on ITRA and UTMB international rankings, the present work offers a more integrative and context-specific understanding of factors potentially influencing sport-specific outcomes. Although the sample size is modest, it represents the largest cohort of internationally ranked female trail runners studied to date, providing valuable reference data on anthropometric and nutritional profiles using validated and replicable methodologies. These findings contribute to the limited evidence available in female endurance sports and may inform applied strategies for performance support and athlete health management.

### 4.1. Nutritional Assessment

The dietary analysis of this cohort of elite female trail runners revealed a widespread prevalence of low energy availability (LEA < 30 kcal/kg) across participants, irrespective of menstrual cycle status. This finding is particularly concerning, given that LEA is the principal etiological factor underlying RED-s [53], a condition associated with impaired performance and adverse long-term health outcomes [54]. Average carbohydrate intake was 4.1 g/kg/day, well below the recommended range for female endurance athletes: 6.0–10.0 g/kg/day [39]. The analysis also highlighted a qualitative imbalance in carbohydrate sources: consumption of complex carbohydrates (e.g., cereals, pasta, rice, bread) was restricted, while the intake of simple carbohydrates (e.g., bars, gels, breakfast cereals) exceeded the recommended threshold of <10% of total energy. This dietary profile suggests a disproportionate reliance on rapidly absorbable, low-fiber carbohydrate sources. Protein intake varied by menstrual status. Athletes with eumenorrheic cycles generally met the recommended intake: 1.4–2.0 g/kg/day [55,56,57], whereas those with amenorrhea/oligomenorrhea exhibited significantly higher intakes (2.5 ± 0.6 g/kg/day), with some individuals reaching values near 3.0 g/kg/day—compatible with hyperproteic diets [54]. This elevated protein intake occurred without a corresponding increase in carbohydrate consumption, and several athletes in the amenorrheic group showed combined patterns of low carbohydrate and high protein intake. Fat intake exceeded recommended levels (20–35% of total caloric intake) in 74.3% of the sample [39]. Nevertheless, the overall quality of fat intake remained within recommended guidelines, with distributions of saturated (5–8%), monounsaturated (15–20%), and polyunsaturated fatty acids (≈5%), and cholesterol intake below 300 mg/day [39]. These values align with previous findings indicating that fat consumption may increase to ~40% of total energy intake during high-volume training periods, such as the general preparatory phase corresponding to the timing of this assessment [58].

Fiber intake was markedly elevated in both groups, consistently surpassing the general recommendation of 25.0–30.0 g/day [59]. While beneficial in moderation, excessive fiber intake may impair the absorption of key minerals, particularly calcium and iron, and contribute to gastrointestinal symptoms such as bloating and flatulence—factors that may hinder athletic performance [60]. However, fiber intake may vary according to the competitive season phase, potentially being reduced during competitions under professional nutritional guidance, which warrants further investigation in different training periods. Vitamin intake was within recommended ranges for all athletes. Although amenorrheic runners reported significantly higher intakes of vitamins B2, B6, C, D, and E, these remained within safe and recommended thresholds [39], indicating overall adequate micronutrient coverage. Mineral intake patterns were similarly favorable for magnesium and zinc, both critical for neuromuscular function and recovery. However, calcium and iron intakes were consistently below recommended levels: 1000.0 mg/day and 18.0 mg/day, respectively [39], potentially compromising bone health, muscle contractility, and oxygen transport—particularly under high training loads and in the presence of menstrual dysfunction [61,62]. This risk may be exacerbated by the aforementioned high fiber intake, which can reduce the bioavailability of these minerals through interference with intestinal absorption [60].

### 4.2. Anthropometric Assessment

The present study provides a detailed anthropometric characterization of elite female trail runners, contributing to the ongoing debate regarding the role of these variables in performance and energy availability. Despite the numerous hypotheses proposed in the literature, the relationship between body composition and sports performance in female endurance athletes—particularly in trail running—remains inconclusive. While some authors have suggested that lower body mass and BMI may be advantageous due to improved cardiorespiratory efficiency [32] and improved race performance [63], other investigations indicate that such advantages may be limited to high-altitude events and are not consistently observed across all race profiles or competitive settings [64]. This variability underscores the multifactorial nature of endurance performance, in which anthropometric traits are likely to interact with physiological, nutritional, psychological, and training-related components. One of the most relevant findings of the present work is the absence of a clearly defined anthropometric profile differentiating athletes based on menstrual status or LEA. Among the 57 anthropometric variables analyzed, only the ectomorphic component demonstrated a statistically significant moderate correlation with LEA (r = 0.418; *p* = 0.012), a magnitude suggesting potential practical relevance, indicating that leaner somatotypes may be more susceptible to energy imbalance. However, this association was moderate, and the limited number of variables showing significant group differences reinforces the notion that no specific somatotype or body composition profile can reliably distinguish athletes with or without menstrual irregularities or LEA in this context. The multivariate analysis, adjusted for body composition, identified a significant overall effect of menstrual status on the anthropometric profile, with discriminant variables including subscapular and abdominal skinfolds, hip and thigh circumferences, and muscle mass estimations. While the Random Forest model achieved high sensitivity in classifying athletes according to menstrual status, its specificity was limited, suggesting that morphological traits alone offer an insufficient predictive framework. Comparison with other published cohorts highlights further complexities. Although our sample shares similar training volumes and competitive achievements with previously studied groups, it stands out for being younger and demonstrating a higher level of international competitiveness. Among the few comparable studies, the one involving elite Colombian runners offers a point of reference [32]. However, clear differences are observed in height, weight, BMI, total skinfold thickness, and somatotype distribution. For example, amenorrheic runners in our study were taller, lighter, and leaner than the Colombian cohort, and also displayed a distinct somatotype profile (endomorphic: 2.2 ± 0.6 vs. 6.5 ± 8.9; mesomorphic: 3.2 ± 1.1 vs. 4.1 ± 0.8; ectomorphic: 3.3 ± 0.9 vs. 2.3 ± 0.9). These differences may be partially explained by ethnic, environmental, and methodological factors, which must be taken into account when drawing comparisons. A further challenge in contextualizing our findings is the lack of methodological consistency across studies. Variations in measurement protocols, body composition estimation equations, and the inconsistent reporting of skinfold data or somatotype values hinder direct comparison and limit the establishment of universal reference standards. In summary, although some morphological patterns were associated with menstrual dysfunction, the overall contribution of anthropometric variables to LEA or performance appears limited in this population. These findings suggest that anthropometric assessments, while informative, should be interpreted alongside physiological, nutritional, and training indicators to better understand performance determinants in elite female trail runners.

### 4.3. Sports Performance Ranking According to Menstrual Cycle

Regarding the influence of menstrual cycle regularity on athletic performance, the results of this study did not reveal statistically significant differences between athletes with regular menstrual cycles and those with irregular cycles in terms of performance scores in long-distance events. Although runners with a regular cycle exhibited slightly higher mean scores and reduced variability, these differences were not substantial enough to reach statistical significance. These findings suggest that, within the analyzed sample, menstrual cycle regularity is not a determining factor for athletic performance. This result is consistent with the existing scientific literature, which generally describes the influence of the menstrual cycle on athletic performance as limited in magnitude and highly variable across individuals. Previous studies have reported that certain phases of the cycle, such as the early follicular phase—characterized by low estrogen and progesterone levels—may be associated with slight reductions in performance due to the limited availability of estrogens, which have known anabolic, antioxidant, and membrane-stabilizing effects [65]. Conversely, phases with higher estrogen concentrations, such as the late follicular phase or ovulation, have been linked to potential improvements in performance, attributed to the hormone’s favorable impact on metabolism and neuromuscular function [65,66]. However, these improvements are not consistently observed across studies and appear to depend on multiple factors, including the type of physical task and individual physiological characteristics. Additionally, the use of hormonal contraceptives—which modulate endogenous hormone levels—has been associated in some studies with a slight reduction in performance. Nevertheless, the reported effects are generally minimal and do not significantly affect most physiological or performance-related parameters [65,67,68,69,70,71].

Taken together, these findings reinforce the notion that menstrual cycle regularity, as a categorical variable, exerts a modest influence on endurance performance. Instead, these results underscore the importance of considering interindividual variability and tailoring training and recovery strategies to the personal experiences, symptoms, and needs of each athlete. Such individualized approaches may be more effective in optimizing performance and well-being in female athletes engaged in high-demand disciplines such as trail and ultra-endurance running.

A potential limitation of this study is the absence of detailed hormonal data in this specific analysis, as this information is addressed in a complementary and independent study currently under review. This separation was intentional to avoid overlap and to allow a deeper interpretation of both physiological and performance aspects. On the other hand, another limitation to consider is that, although the 7-day food diary and 24 h recall methods used are generally considered robust for capturing dietary intake variability in athletes, self-reported dietary assessments are prone to reporting bias (e.g., under- or over-reporting), which may affect the accuracy of nutrient intake estimates despite the use of visual portion-size guides and individualized instructions provided to participants. Regarding the estimation of energy expenditure and energy availability, the study used the Harris-Benedict equation with a fixed activity factor and standard MET values. This approach did not account for individual variations in exercise intensity, specific terrain characteristics in trail running, or personal metabolic differences among the athletes. This simplification, inherent to the study design, may limit the accuracy of energy expenditure and availability estimates, highlighting an area for improvement in future studies incorporating more direct and personalized measurements.

Finally, due to the cross-sectional design, it is difficult to ascertain whether the low energy availability observed represents an adaptive condition or a problematic one, since information about the chronicity and duration of physiological adaptations is lacking. Some athletes presented menstrual disturbances and a history of stress fractures, indicating potential problematic low energy availability. This limitation restricts the full application of the recent 2023 IOC consensus definitions on RED-s differentiating adaptive versus problematic low energy availability [72,73]. Therefore, results and conclusions should be interpreted cautiously, emphasizing the need for prospective studies to enable a more detailed and dynamic assessment of low energy availability in female athletes.

## 5. Conclusions

This study underscores the multifactorial nature of health and performance in elite female trail runners. Despite generally adequate vitamin intake, a high prevalence of LEA, insufficient carbohydrate, calcium, and iron intake, and excessive fiber consumption may compromise both physiological function and performance. These imbalances—often combined with high-protein and high-fat dietary patterns— emphasize the need for individualized nutritional strategies that, according to the findings of this study, ensure sufficient energy availability, prioritize complex over simple carbohydrates, and optimize micronutrient intake—particularly calcium, iron, and vitamins D and B-complex—to support metabolic and hormonal balance. No significant differences in performance or anthropometric profiles were found between athletes with or without menstrual dysfunction, although a moderate association emerged between an ectomorphic profile and LEA. The results challenge reductionist approaches based solely on anthropometry and support a more integrated perspective. Practically, regular nutritional screening and targeted education programs are essential to help female trail runners avoid LEA and the broader RED-S syndrome, maintaining systemic health as the foundation for sustainable, long-term athletic performance. The performance assessment, while comprehensive, may favor participation frequency over peak outcomes. Limitations include the cross-sectional design, self-reported dietary data, and the absence of hormonal profiling in this analysis. Future longitudinal and multidisciplinary studies are needed to better understand risk factors and inform preventive strategies in this population.

## Figures and Tables

**Table 1:** Chronological age and training characteristics of mountain runners according to whether they report menstrual cycle dysfunctions or not.

	Total Group (*n* = 35)	Eumenorrhea (*n* = 12)	Secondary Amenorrhea/Oligomenorrhea (*n* = 23)	Difference (%)	*t*-Test (*p*)	Effect-Size (*d*)
Age (years)	33.7 ± 7.5	37.1 ± 6.5	31.9 ± 7.5	15.6	−2.049 (0.048) *	0.69
Training volume (h/week)	12.9 ± 3.7	12.7 ± 3.1	13.0 ± 4.0	2.3	0.219 (0.828)	0.08
Previous experience (years)	8.4 ± 5.1	9.9 ± 4.9	7.6 ± 5.1	27.1	−1.263 (0.216)	0.45
Competitive ranking (ITRA-UTMB points)	656.9 ± 63.6	663.8 ± 36.7	653.3 ± 74.5	1.6	−0.554 (0.583)	0.17

Values are expressed as mean ± standard deviation. ITRA-UTMB: International Trail Running Association–Ultra-Trail du Mont-Blanc performance index. ES: Effect Size (Cohen’s d); * indicates statistically significant differences (*p* < 0.05); *p*: Statistical significance level (*p*-value).

**Table 2:** Nutritional status of female mountain runners according to whether they report menstrual cycle dysfunctions or not.

		Whole Group (*n* = 35)	Eumenorrhea (*n* = 12)	Secondary Amenorrhea/Oligomenorrhea (*n* = 23)	Difference (%)	*t*-Test (*p*)	Effect-Size (*d*)
Energy availability	Ingestion (kcal)	2041 ± 199.2	2032.4 ± 222.1	2045.5 ± 191.2	0.6	1.876 (0.069)	0.06
	Expenditure (kcal)	2888.5 ± 145.5	2961.0 ± 137.4	2850.7 ± 137.5	3.8	−2.254 (0.031) *	0.80
	Availability (kcal/kg)	23.7 ± 5.1	21.5 ± 3.9	24.8 ± 5.3	13.8	0.182 (0.856)	0.71
Carbohydrates	Total Carbohydrates (g)	211.7 ± 40.1	225.5 ± 48.3	204.5 ± 34.1	9.9	−1.497 (0.144)	0.50
	Total Carbohydrates (%)	41.4 ± 6.0	43.7 ± 4.5	40.1 ± 6.5	8.6	−1.708 (0.097)	0.64
	Total Carbohydrates (g/kg/day)	4.1 ± 0.6	4.1 ± 0.7	4.1 ± 0.6	0.2	0.044 (0.965)	0.01
	Complex Carbohydrates (g)	98.7 ± 38.4	129.7 ± 27.0	82.5 ± 33.3	47.9	−4.236 (<0.001) *	1.56
	Complex Carbohydrates (%)	19.1 ± 6.7	25.6 ± 4.2	15.8 ± 5	51.5	−5.850 (<0.001) *	2.12
	Simple Carbohydrates (g)	104.1 ± 32.2	90.9 ± 23.4	110.9 ± 34.5	19.3	1.802 (0.081)	0.69
	Simple Carbohydrates (%)	20.2 ± 5.4	17.2 ± 3.1	21.8 ± 5.7	23.0	2.616 (0.014) *	1.00
Proteins	Total Proteins (g)	111 ± 25	96.2 ± 11.8	118.7 ± 26.7	20.3	3.448 (0.002) *	1.09
	Total Proteins (%)	21.9 ± 4.7	19.1 ± 1.4	23.3 ± 5.1	19.2	3.664 (0.001) *	1.12
	Total Proteins (g/kg/day)	2.2 ± 0.6	1.7 ± 0.2	2.5 ± 0.6	34.4	5.956 (<0.001) *	1.79
Lipids and fats	Total Lipids (g)	81.7 ± 7.4	81.7 ± 6.6	81.7 ± 7.9	0.1	−0.033 (0.974)	0.01
	Total Lipids (%)	36.7 ± 3.9	36.7 ± 5.4	36.7 ± 3.0	0.2	−0.039 (0.969)	0.01
	Total Lipids (g/kg/day)	1.6 ± 0.2	1.5 ± 0.1	1.7 ± 0.2	10.5	3.116 (0.004) *	1.26
	Saturated Fats (%)	10.1 ± 2.4	12.3 ± 1.3	8.9 ± 2.0	33.9	−6.199 (<0.001) *	2.02
	Monounsaturated Fats (%)	17.7 ± 4.3	21.6 ± 5.1	15.7 ± 1.8	33.6	−3.917 (<0.001) *	1.54
	Polyunsaturated Fats (%)	7.3 ± 1.2	7.6 ± 1.7	7.1 ± 0.9	7.5	−1.060 (0.296)	0.37
	Cholesterol (mg)	298.9 ± 83.3	305.1 ± 37.9	295.7 ± 99.9	3.2	−0.403 (0.689)	0.12
Fiber	Fiber (g)	36.3 ± 13.7	30.4 ± 7.6	39.4 ± 15.3	24.7	2.316 (0.027) *	0.74
Vitamins	Vitamin B1 (mg)	2.1 ± 0.6	2.0 ± 0.6	2.1 ± 0.6	4.8	0.489 (0.628)	0.16
	Vitamin B2 (mg)	1.9 ± 0.4	1.7 ± 0.3	2.0 ± 0.5	18.5	2.410 (0.021) *	0.72
	Vitamin B6 (mg)	2.8 ± 0.8	2.3 ± 0.2	3.1 ± 0.8	29.4	4.464 (<0.001) *	1.37
	Vitamin B12 (mcg)	4.4 ± 1.4	5.0 ± 1.4	4.1 ± 1.3	20.6	0.666 (0.510)	0.66
	Vitamin C (mg)	249.7 ± 125	178.3 ± 52.0	286.9 ± 136.2	43.5	3.379 (0.002) *	1.05
	Vitamin D (mcg)	4.9 ± 2.5	3.5 ± 1.4	5.6 ± 2.6	43.4	2.619 (0.014) *	1.01
	Vitamin E (mg)	15.3 ± 3.7	12.3 ± 1.4	16.8 ± 3.6	30	5.405 (<0.001) *	1.65
Minerals	Ca (mg)	879.1 ± 206.4	762.2 ± 130.1	940.1 ± 214.5	20.2	2.621 (0.014) *	1.00
	Fe (mg)	16.6 ± 3.2	15.3 ± 2.6	17.3 ± 3.4	11.9	1.774 (0.085)	0.66
	Mg (mg)	465.8 ± 108.3	424.8 ± 90.9	487.2 ± 112.3	13.4	1.660 (0.106)	0.61
	Zn (mg)	10.8 ± 1.8	11.2 ± 1.7	10.5 ± 1.9	6.9	−1.133 (0.267)	0.39

Values are expressed as mean ± standard deviation. Energy availability: ingestion and expenditure refer to kilocalories (kcal), and availability is reported in kcal/kg of fat-free mass. Macronutrient intake is reported in grams (g), percentage of total energy intake (%), or grams per kg of body weight per day (g/kg/day). Vitamin and mineral intakes are expressed in mg, µg, or mg/day as appropriate. ES: Effect Size (Cohen’s d); * indicates statistically significant differences (*p* < 0.05); *p*: Statistical significance level (*p*-value).

**Table 3:** Anthropometric profile (ISAK protocol) of mountain runners according to the presence or absence of menstrual cycle dysfunctions.

		Whole Group (*n* = 35)	Eumenorrhea (*n* = 12)	Secondary Amenorrhea/Oligomenorrhea (*n* = 23)	Difference (%)	*t*-Test (*p*)	Effect-Size (*d*)
Base measures	Body mass (kg)	52.7 ± 3.8	54.3 ± 2.5	52.0 ± 4.1	4.4	−1.752 (0.089)	0.61
	Stretch stature (cm)	162.7 ± 4.2	163.0 ± 3.0	162.6 ± 4.7	0.2	−0.235 (0.816)	0.10
	Sitting height (cm)	86.5 ± 2.5	86.6 ± 1.9	86.4 ± 2.8	0.3	−0.307 (0.761)	0.08
	Arm span (cm)	163.2 ± 5.9	163.6 ± 4.2	163.1 ± 6.8	0.3	−0.248 (0.806)	0.08
Skinfolds (mm)	Triceps	10.9 ± 2.6	11.6 ± 2.5	10.6 ± 2.7	9.5	−1.109 (0.275)	0.38
	Subscapular	6.2 ± 1.4	7.0 ± 1.1	5.9 ± 1.3	17.7	−2.433 (0.020) *	0.79
	Biceps	3.7 ± 1.2	3.8 ± 0.8	3.7 ± 1.4	1.2	−0.102 (0.919)	0.08
	Iliac crest	9.2 ± 2.9	9.3 ± 3.4	9.1 ± 2.7	2.3	−0.196 (0.846)	0.07
	Supraspinale	5.1 ± 1.4	5.5 ± 1.0	4.9 ± 1.6	10.9	−1.106 (0.277)	0.43
	Abdominal	9.5 ± 3.9	11.6 ± 4.5	8.4 ± 3.1	33.8	−2.469 (0.0192) *	0.82
	Thigh	15.7 ± 4.8	16.5 ± 5.4	15.3 ± 4.5	7.6	−0.696 (0.491)	0.25
	Calf	6.4 ± 2.3	6.7 ± 2.6	6.2 ± 2.1	7.8	−0.607 (0.548)	0.22
Girths (cm)	Head	54.3 ± 1.2	54.6 ± 1.5	54.1 ± 1.1	0.8	−1.037 (0.307)	0.42
	Neck	30.4 ± 1.9	31.1 ± 2.9	30.1 ± 1.1	3.3	−1.044 (0.304)	0.53
	Arm relaxed	24.4 ± 1.7	25.1 ± 1.7	24.1 ± 1.7	4.1	−1.654 (0.108)	0.59
	Arm flexed and tensed	25.8 ± 1.7	26.5 ± 1.8	25.5 ± 1.6	3.7	−1.585 (0.122)	0.59
	Forearm	22.2 ± 1.0	22.2 ± 1.0	22.2 ± 1.1	0.3	0.160 (0.874)	0.01
	Wrist	14.2 ± 0.6	14.1 ± 0.4	14.2 ± 0.7	0.8	0.512 (0.612)	0.17
	Chest	81.9 ± 3.2	82.4 ± 2.6	81.6 ± 3.5	1.0	−0.681 (0.501)	0.25
	Waist	66.0 ± 2.9	66.1 ± 1.8	65.9 ± 3.3	0.4	−0.302 (0.764)	0.07
	Hip	89.3 ± 3.5	91.2 ± 3.0	88.3 ± 3.4	3.3	−2.537 (0.016) *	0.83
	Thigh 1 cm gluteal	52.1 ± 2.6	53.3 ± 2.4	51.5 ± 2.5	3.4	−2.039 (0.049) *	0.69
	Thigh middle	47.2 ± 1.9	48.0 ± 1.4	46.7 ± 2.0	2.6	−1.926 (0.063)	0.68
	Calf	33.4 ± 1.4	34.1 ± 1.0	33.1 ± 5.0	3.2	−2.244 (0.032) *	0.71
	Ankle	20.6 ± 1.2	20.4 ± 1.0	20.7 ± 1.3	1.6	0.777 (0.443)	0.25
Lengths and heights (cm)	Acromiale-radiale	31.3 ± 1.2	31.4 ± 1.1	31.2 ± 1.3	0.6	−0.462 (0.647)	0.17
	Radiale-stylion	23.8 ± 1.1	23.8 ± 1.4	23.8 ± 1.0	0.1	−0.065 (0.948)	0.01
	Midstylion-dactylion	18.3 ± 0.9	18.2 ± 0.6	18.3 ± 1.0	0.8	0.561 (0.578)	0.11
	Iliospinale height	90.4 ± 2.7	90.3 ± 2.3	90.4 ± 3.0	0.1	0.080 (0.937)	0.04
	Trochanterion height	85.2 ± 3.4	85.4 ± 3.1	85.2 ± 3.6	0.2	−0.168 (0.868)	0.06
	Trochanterion-tibiale laterale	43.1 ± 3.1	42.5 ± 3.6	43.5 ± 2.7	2.1	0.848 (0.402)	0.32
	Tibiale laterale height	42.6 ± 2.0	43.1 ± 1.4	42.3 ± 2.2	1.9	−1.158 (0.255)	0.40
	Foot	36.2 ± 1.4	36.2 ± 1.2	36.2 ± 1.5	0.2	0.149 (0.882)	0.01
	Tibiale mediale-sphyrion tibiale	24.4 ± 1.0	24.2 ± 0.8	24.5 ± 1.1	1.3	0.897 (0.376)	0.30
Breadths (cm)	Biacromial	35.3 ± 2.0	35.8 ± 1.1	35.1 ± 2.4	1.8	−0.896 (0.377)	0.35
	Antero-posterior abdominal depth	16.7 ± 1.7	17.2 ± 1.4	16.4 ± 1.8	4.5	−1.254 (0.219)	0.47
	Biiliocrestal	26.3 ± 2.6	26.5 ± 3.5	26.3 ± 2.0	1.0	−0.281 (0.780)	0.08
	Transverse chest	24.0 ± 2.1	25.2 ± 1.4	23.4 ± 2.1	7.5	−2.640 (0.012) *	0.86
	Antero-posterior chest depth	16.5 ± 1.8	16.6 ± 1.4	16.5 ± 1.9	0.7	−0.178 (0.860)	0.06
	Humerus	6.0 ± 0.3	6.1 ± 0.2	6.0 ± 0.3	1.6	−0.999 (0.325)	0.33
	Bi-styloid	4.9 ± 0.2	4.9 ± 0.2	4.9 ± 0.2	1.3	−0.820 (0.418)	0.01
	Femur	8.8 ± 0.4	8.9 ± 0.3	8.7 ± 0.4	2.8	−1.880 (0.069)	0.50
	Bimalleolar	6.4 ± 0.4	6.3 ± 0.5	6.4 ± 0.3	1.2	0.541 (0.592)	0.25
Proportionality and muscle areas	BMI (kg/m^2^)	19.9 ± 1.3	20.4 ± 0.9	19.7 ± 1.4	3.5	−1.575 (0.125)	0.54
	WHR (cm/cm)	0.74 ± 0.1	0.73 + 0.1	0.75 + 0.1	2.9	1.621 (0.114)	0.20
	CMO (g/g)	3.8 ± 1.0	3.6 ± 0.4	4.0 ± 1.2	11.5	1.225 (0.230)	0.44
	Arm muscle area (cm^2^)	28.8 ± 5.9	30.3 + 5.8	28.0 + 5.9	7.8	−1.091 (0.283)	0.39
	Thigh muscle area (cm^2^)	135.6 ± 12.1	139.3 + 9.2	133.7 + 13.1	4.1	−1.307 (0.200)	0.46
	Calf muscle area (cm^2^)	72.1 ± 7.3	75.1 + 6.0	70.6 + 7.5	6.2	−1.782 (0.084)	0.62
Body composition	∑8 skinfolds (mm)	66.8 ± 14.7	72.0 ± 15.7	64.1 ± 13.8	11.8	−1.529 (0.136)	0.54
	∑6 skinfolds (mm)	53.9 ± 11.8	58.9 ± 12.8	51.3 ± 10.6	14.1	−1.875 (0.070)	0.64
	Muscle Mass-Lee (%)	40.3 ± 2.0	39.9 ± 2.3	40.5 ± 1.8	1.5	0.841 (0.406)	0.30
	Fat Mass-Withers (%)	12.6 ± 2.6	13.7 ± 2.2	12.1 ± 2.7	12.5	−1.737 (0.092)	0.62
	Bone Mass-Rocha (%)	16.8 ± 1.2	16.7 ± 1.0	16.9 ± 1.3	1.3	0.552 (0.585)	0.17
Somatotype	Endomorphy	2.3 ± 0.6	2.5 ± 0.5	2.2 ± 0.6	13.6	−1.598 (0.120)	0.50
	Mesomorphy	3.4 ± 1.0	3.9 ± 0.6	3.2 ± 1.1	18.7	−1.917 (0.064)	0.70
	Ectomorphy	3.2 ± 0.8	3.0 ± 0.5	3.3 ± 0.9	10.2	1.206 (0.237)	0.38

Values are expressed as mean ± standard deviation. BMI: Body Mass Index (kg/m^2^); WHR: Waist-Hip Ratio (cm/cm); SMM: Skeletal Muscle Mass (%); FM: Fat Mass (%); BMC: Bone Mineral Content (%); ES: Effect Size (Cohen’s d); * indicates statistically significant differences (*p* < 0.05); *p*: Statistical significance level (*p*-value).

## Data Availability

Data availability will be available after acceptance and publishing in this journal.

## Abbreviations

The following abbreviations are used in this manuscript:

FDR	False Discovery Rate
ISAK	International Society for the Advancement of Kinanthropometry
ITRA	International Trail Running Association
LDA	Linear Discriminant Analysis
LEA	Low Energy Availability
LEAF-Q	Low Energy Availability in Females Questionnaire
PCA	Principal Component Analysis
RED-s	Relative Energy Deficiency in sport
TR	Trail Running
UTMB	Ultra-Trail Mont-Blanc
VIF	Variance Inflation Factor
